# Complications, burden and in-hospital death among hospital treated injury patients in Victoria, Australia: a data linkage study

**DOI:** 10.1186/s12889-019-7080-y

**Published:** 2019-06-21

**Authors:** Dasamal Tharanga Fernando, Janneke Berecki-Gisolf, Stuart Newstead, Zahid Ansari

**Affiliations:** 1 Monash University Accident Research Centre, Monash University, Clayton Campus, 21 Alliance Lane, Clayton, Victoria 3800 Australia; 2 Victorian Agency for Health Information, 50 Lonsdale Street, Melbourne, Victoria 3000 Australia

**Keywords:** Burden, Complications, Death, Hospital-admitted, Injury, Linkage, Outcome, Readmission

## Abstract

**Background:**

A wide range of outcome measures can be calculated for hospital-treated injury patients. These include mortality, use of critical care services, complications, length of stay, treatment costs, readmission and nursing care after discharge. Each address different aspects and phases of injury recovery and can yield vastly different results. This study aims to: (1) measure and report this range of outcomes in hospital-treated injury patients in a defined population; and (2) describe the associations between injury characteristics, socio-demographics and comorbidities and the various outcomes.

**Methods:**

A retrospective analysis was conducted of injury-related hospital admissions from July 2012 to June 2014 (152,835 patients) in Victoria, Australia. The admission records were linked within the dataset, enabling follow-up, to assess the outcomes of in-hospital death, burden, complications and 30-day readmissions. Associations between factors and outcomes were determined using univariate regression analysis.

**Results:**

The proportion of patients who died in hospital was 0.9%, while 26.8% needed post-discharge care. On average patients had 2.4 complications (confidence interval (CI) 2.4–2.5) related to their initial injury, the mean cost of treating a patient was Australian dollars 7013 (CI 6929–7096) and the median length of stay was one day (inter quartile range 1–3). Intensive-care-unit-stay was recorded in 3% of the patients. All-cause 30-day readmissions occurred in 12.3%, non-planned 30-day readmissions in 7.9%, while potentially avoidable 30-day readmissions were observed in 3.2% of the patients. Increasing age was associated with all outcomes. The need for care post-discharge from hospital was highest among children and the oldest age group (85 years and over). Injury severity was associated with all adverse outcomes. Increasing number of comorbidities increased the likelihood of all outcomes. Overall, outcomes are shown to differ by age, gender, comorbidities, body region injured, injury type and injury severity, and to a lesser extent by socio-economic areas.

**Conclusions:**

Outcomes and risk factors differ depending on the outcome measured, and the method used for measuring the outcome. Similar outcomes measured in different ways produces varying results. Data linkage has provided a valuable platform for a comprehensive overview of outcomes, which can help design and target secondary and tertiary preventive measures.

**Electronic supplementary material:**

The online version of this article (10.1186/s12889-019-7080-y) contains supplementary material, which is available to authorized users.

## Background

Injury hospitalisation rates have risen in Australia at 1% per year [1, 2] and in 2011, injury was ranked fifth among the most common causes for disease burden; burden was measured as years lost to premature death or living with illness [3]. Injury can be life-threatening, lead to permanent disability, or impair functional outcomes, resulting in poor quality of life. Adverse injury outcomes include accelerated mortality [4–8], development of complications [9–11], additional use of critical care services (CCS) during hospital stay [10, 12–14], need for long-term nursing care post-hospital-discharge [12, 15, 16], extended length of hospital stay (LOS) [8, 17], increased hospital costs [14, 18] and increased likelihood of readmission to hospital (post-discharge for an index condition) [8, 19].

Various methods exist for measuring each of these injury outcomes. Mortality following hospitalisation can be examined at different time points, including death in hospital [4, 5, 17, 20–22], 30-day mortality [23], death within one year [7, 17, 22] or time until death [24, 25], while readmission can be measured as all-cause [8, 26], non-planned [19, 27] or potentially avoidable [26, 28–30]. Similarly, complications can be identified as the leading adverse event (using a hierarchy of codes for ranking importance) [31], the presence of at least one adverse event [32] or count of multiple adverse events [33]. Complications can also be distinguished as hospital acquired [32, 34] or non-hospital acquired (i.e., complications related to the injury or comorbidity). Injury outcomes can vary depending on the aspect studied: for example, *all-cause* 30-day readmissions are much more common than *potentially avoidable* 30-day readmissions [26]. Similarly, *in-hospital* mortality is much lower than *one-year* mortality [7, 17, 22]. Although each of these listed measures are quantifications of injury outcomes, they are rarely used in combination and compared, even though outcomes (and risk factors for adverse outcomes) can differ based on which measure is used. Reporting of injury outcomes and risk factors will help with design of injury outcome studies, and interpretation and comparison of published injury outcomes studies conducted in similar settings.

Numerous studies have established that outcomes are associated with factors such as age [5, 8, 11, 35] and gender [23, 36]. Among injury patients, injury mechanism [4, 6, 11, 14] and severity [4, 6, 14, 37] play a key role. The presence of pre-existing chronic diseases/conditions (comorbidities) can also have negative effects on injury outcomes [5–8, 12, 38, 39].

In 2015–16, there were around 10.6 million hospital separations (episodes of admitted care) from public and private hospitals in Australia and a quarter of them were from hospitals in Victoria, Australia’s second most populous state [40]. The primary aim of this paper is to describe a range of injury outcomes among patients admitted to Victorian hospitals. The outcomes are: complications, 30-day readmissions, LOS, hospital costs, use of CCS, discharge destination and in-hospital death. A secondary aim is to establish the association between each outcome and patient age and sex, body region injured, injury type, injury severity and the presence of comorbidities.

## Methods

A retrospective analysis was carried out on existing morbidity data of hospital admitted injury-patients in Victoria; the hospital data was internally linked to enable follow up of cases, in terms of subsequent admissions and in-hospital death. Data provision and linkage was undertaken by the Centre for Victorian Data Linkage (CVDL). Ethics approval was obtained from the Monash University Human Research Ethics Committee.

### Data sources

Morbidity data was extracted from the Victorian Admitted Episodes Dataset (VAED), which contains unit records of all public and private hospital admissions in Victoria. Among the information stored in the VAED are patient demographics, morbidity, external causes of morbidity and patient discharge status. Up to forty diagnosis codes can be recorded per patient episode; this includes external cause information. Morbidity information in the VAED is coded according to the International Statistical Classification of Diseases and Related Health Problems, Tenth Revision, Australian Modifications (ICD-10-AM) [41]. Treatment costs at hospitals for each episode were extracted from the Victorian Cost Data Collection (VCDC), these represent the total actual costs of the episodes as reported by the hospital.

### Data linkage

VAED hospital admissions data were internally linked within the VAED and linked with VCDC data using variables such as campus code, unique record number and department of health key. Linkage within the VAED was enhanced with the Better Patient Data dataset (which is a separate collection from clinical data, containing identifying information such as patients’ names, addresses, sex, date of birth etc.) in order to create across-service patient records. CVDL used deterministic data linkage with some fuzzy matching to allow for slight variation in the linkage variables, such as incorrect names and dates. No clerical review of linkages was carried out but a number of general quality checks were performed by CVDL.

### Case selection

Injury cases were selected using a protocol established for previous national reporting in Australia [1]: VAED records with an injury diagnosis (ICD-10-AM diagnosis code in the range “S00” to “T75” or “T79” in the principal diagnosis code). The selected cases were Victorian residents with their index injury hospital admission during the study period. ‘Index injury’ is the first injury record in the VAED for a patient during that period. Records with an admission source indicating a transfer from another hospital or statistical separation (change in care type within the same hospital) in consecutive records were all considered to be part of the index injury hospitalisation.

Patients with an index injury admission between 01 July 2012 and 30 June 2014 in the VAED were selected. Using internally linked data within the VAED (i.e. using a patient-specific identifier), patients were followed up for all hospital admissions subsequent to their index injury admission. The follow-up times were varied by outcome: one year for in-hospital death and cost, two years for LOS, discharge destination, intensive care unit (ICU) stay and mechanical ventilator (MV) use, and thirty days for readmissions, from the index injury date.

### Outcome coding and case exclusions

#### In-hospital death

In-hospital death was classified as a binary outcome and excluded those whose index stay was longer than 365 days.

#### Readmissions

Three different forms of readmissions were coded, all with a binary outcome: “1” for patients readmitted within 30 days of index admission discharge and “0” otherwise, limited to the first occurring readmission. The three forms were: 1) at least one *any-cause* readmission; 2) at least one *non-planned* readmission (elective admissions were excluded); and 3) at least one *potentially avoidable* readmission.

Potentially avoidable readmissions in this study were identified using two methods. First, by selecting non-planned readmission records with any primary condition diagnosis code equal to the principal diagnosis code of the index admission. Primary conditions were identified with a prefix “P” in the VAED, which means they existed at the time of hospital admission and were treated or actively monitored during the stay. Second, by using part of the SQLape (Striving for Quality Level and Analyzing of Patient Expenses) algorithm [42]. The algorithm identifies potentially avoidable readmissions as unforeseen readmissions for a previously known affliction. They consist of iatrogenic complications, other healthcare related complications and complications arising from preventable diseases (deep vein thrombosis, pulmonary embolism, decubitus ulcer). In this study, following this algorithm, readmissions with an SQLape listed complication recorded as a primary condition were selected (these complication codes are listed in appendix D of Halfon et al. (2002) [43]). Certain complications from this list were not included in identifying potentially avoidable readmissions as they were irrelevant to the scope of studying injury patients; namely complications related to obstetrics and radiation-induced conditions. Codes related to complications arising from pre-existing diseases were also not considered in identifying potentially avoidable readmissions. The reason being, by including readmissions related to pre-existing conditions, an effect of these conditions becomes inherent in the outcome, whereas the study aims to describe the effect of pre-existing conditions on the outcome. Patients who died in hospital or left against medical advice were excluded from ‘readmissions’ coding.

#### Complications

Complications were identified using the Classification of Hospital Acquired Diagnoses (CHADx) [32], a tool developed to code hospital-acquired diagnoses, and commonly used in hospitals in a number of Australian states. The CHADx uses a “condition onset” flag which distinguishes complications from primary diagnoses and comorbidities. The CHADx is grouped into 17 major classes expanding to 144 subclasses and is designed to avoid double counting in sequentially coded diagnosis information. Complications were determined for all index admissions and related readmissions (i.e., readmissions with the same principal diagnosis code as the index principal diagnosis code or a principal readmission diagnosis code of T79, T80-T89 or T90-T98; including complication codes recorded in transfers and statistical separations records). Readmissions that took place later than six months after the index admission were excluded. All complications were computed according to the CHADx hierarchy and summed as a count variable (number of complications).

#### Costs and LOS

Costs were recorded in Australian dollars (AUD), and LOS (excluding leave with and without permission) in days. The outcomes were cost and LOS of the index admission plus statistical separations and transfers to other hospitals subsequent to the index admission, and readmissions were excluded. Cost data were available for July 2012 to June 2015 allowing only one-year follow-up for patients who entered hospitals after June 2014. For this reason, cost analysis was restricted to patients with a length of stay equal to or less than one year. Cost data was available only for public hospitals and some public hospital patient records had missing data. Therefore, the cost data analysis was limited to patients admitted to public hospitals with non-missing cost data. This amounted to 77.6% of the index admission cohort; the remaining 22.4% being index injury admissions to private hospitals, patients who stayed more than one year in public hospitals with non-missing data and those with missing cost data for public hospitals. Costs were standardized to 2012 prices using the Australian Consumer Price Index [44].

#### Discharge destination

Long-term nursing care needs were assessed based on post-hospital discharge destination. Discharge destination was classified into a binary variable with patients either (1a) discharged home with a specific referral for further care to a support service, (1b) transferred to aged care or mental health residential facility or (1c) transferred to other hospital-based care, vs (2) sent home without a referral for further care. Patients who left against medical advice and those who died in hospital were excluded. Further, patients were retained only if the admission source was private residence or accommodation. This step excluded patients transferred from transition care bed-based programs, mental health residential facilities and aged care residential facilities. These patients were excluded as they are most likely to go back to accommodation ‘with care’ regardless of the injury and demographic factors.

#### Critical care services

CCS analysed in this study were ICU stay, cardiac care unit (CCU) stay and MV use. CCS were modelled as a binary response.

### Factor coding and grouping

Patients were classified into six age groups, closely aligned to the groups used by the Australian Bureau of Statistics [45]; 0–14, 15–24, 25–44, 45–64, 65–84, 85 years and over. Body region groups were created by regrouping the “blocks” contained in the ICD-10-AM Chapter 19 [41] and injury groups according to the injury “type” classification at the three-character level in the same chapter; both based on the principal diagnosis code. Injury severity was calculated using the ICD-based Injury Severity Score (ICISS) [46]. Computation of the ICISS was as follows. First, each injury diagnosis in every record was allocated a survival risk ratio (SRR) (proportion of survivors among all patients with that particular ICD-10-AM diagnosis); SRRs were sourced from the National Injury Surveillance Unit [47]. Next, using the *worst injury* method [48], the lowest SRR was assigned as the ICISS. Finally, a serious injury was considered to be one with an ICISS less than or equal to 0.941 (survival probability of 94.1%) [49]. Socio economic status was classified as per the Socio-Economic Indexes for Areas (SEIFA) [50]. The specific SEIFA used in this study was the Index of Relative Socio-Economic Advantage and Disadvantage (IRSAD); with state deciles based on Statistical Local Areas (SLAs). A low score indicates relatively greater disadvantage. Comorbidities were identified using a combination of the Charlson Comorbidity Index (CCI) [51] and Elixhauser Comorbidity Measure (ECM) [52] comorbidity groups. The ICD-10 codes for these conditions were extracted mainly from Quan et al. (2005) [53] and Sundararajan et al. [54]. This included all 17 conditions from the CCI list and all 30 conditions from the ECM list. Thirty-four comorbidity groups were identified in total by combining these two lists, of which three were eventually excluded. The excluded groups were weight loss and fluid-electrolyte disorders, which were more likely to be complications or symptoms, rather than comorbidities, and the group “other neurological disorders” which were a diverse selection of codes that did not narrow down to a specific condition. Additional codes from the Sundararajan list included the following: B23 (HIV/AIDS), C80 (any malignancy), R02 (peripheral vascular disease), N01, N072-N074 (renal disease).

### Statistical analysis

Descriptive analysis was undertaken to determine the distribution of outcomes across selected demographic and patient characteristics of the study population; these were captured as frequencies and proportions for binary outcomes, means with 95% confidence intervals (CIs) and medians with inter-quartile ranges (IQRs) for continuous variables.

Sample sizes were large in this study, therefore effect sizes were more meaningful than *p* values to establish associations between outcomes and population factors [55]. Univariate modelling was carried out using: (i) logistic regression for discharge destination, in-hospital death, CCS use and readmissions, (ii) negative binomial regression for LOS and number of complications and (iii) linear regression with a natural log transformation for costs. The aim of this study was to describe outcomes and their association with factors, therefore establishing associations between outcomes and base factors using univariate analysis was adequate. Additional analysis for associations between outcomes were also carried out using univariate modelling. Once again using simple tests for associations such as the chi squares were not possible with this large dataset, because all results are highly significant and therefore does not provide further insights. Association between the dependent and independent variables were assessed based on effect sizes (using odds ratios (ORs) for logistic regressions, incident rate ratios (IRRs) for negative binomial regressions and beta coefficients for linear regressions, and their confidence intervals); with an effect size of ±30% considered as notable. The normative (logical) or largest categories were used as reference groups for regression analysis. SAS software, Version 9.4 of the SAS System for Windows [56] and Stata 14.0 (StataCorp) [57] were used to analyse the data.

## Results

There were 152,835 patients with an index hospital admission for an injury during the period July 2012 to June 2014. Males represented a higher proportion (55.8%), while patients with serious injury (ICISS less than or equal to 0.941) accounted for 12.5% and those with one or more comorbidities accounted for 19.5%. The body regions most commonly injured were the head (17.0%) and wrist and hand (16.8%) while fractures were the most common type of injury (41.6%) (Table 1).

**Table 1:** Overview of study population: hospital-admitted injury patients, Victoria

	Injury hospital admisions^a^	
	July 2012 to June 2014	
Total number of patients	152,835	
Age group (years)		
0–14 years	21,058	13.8
15–24 years	22,851	15.0
25–44 years	35,394	23.2
45–64 years	29,325	19.2
65–84 years	27,886	18.3
85 and over	16,321	10.7
Gender		
Male^b^	85,329	55.8
Female	67,506	44.2
Injury severity^c^		
Serious injury (ICISS< 0.941)	19,095	12.5
Other injury (ICISS> = 0.941)	133,740	87.5
Number of comorbidities		
0	123,046	80.5
1	21,046	13.8
2	5699	3.7
3	2120	1.4
> 3	924	0.6
Body region		
Head	25,982	17.0
Neck	3428	2.2
Thorax	7359	4.8
Abdomen, lower back, lumbar spine & pelvis	9410	6.2
Shoulder & upper arm	11,224	7.3
Elbow & forearm	15,465	10.1
Wrist & hand	25,722	16.8
Hip & thigh	12,862	8.4
Knee & lower leg	19,806	13.0
Ankle & foot	5662	3.7
Foreign body (various regions)	2585	1.7
Burns (various regions)	1802	1.2
Multiple body regions	36	0.0
Unspecified body region	534	0.4
Body region not relevant	10,958	7.2
Grouped injury type (first occurring)		
Superficial injury	7364	4.8
Open wound	21,309	13.9
Fracture	63,505	41.6
Dislocation, sprain & strain	10,502	6.9
Injury to nerves & spinal cord	1990	1.3
Injury to blood vessels	1332	0.9
Injury to muscle & tendon	8205	5.4
Crushing injury	326	0.2
Traumatic amputation	1588	1.0
Eye injury- excluding foreign body	481	0.3
Intracranial injury	6038	4.0
Injury to internal organs	1684	1.1
Foreign body	2585	1.7
Burns	1802	1.2
Other and unspecified injury	13,166	8.6
Systemic-poisoning/toxic effects	9782	6.4
Other effects of external cause/complication	1176	0.8
Socio-Economic Indexes for Areas (SEIFA)^d^		
1	16,548	10.8
2	#	#
3	9982	6.5
4	16,954	11.1
5	10,147	6.6
6	11,217	7.3
7	11,123	7.3
8	24,412	16.0
9	22,627	14.8
10	21,648	14.2
999 (missing)	< 5	< 5

Notes:

^a^Total number of patients with an index admission to all public and private hospitals in Victoria, limited to Victorian residents with an ICD-10-AM diagnosis code in the range “S00” to “T75” or “T79” in the principal diagnosis

^b^Intersex patient count less than 3 added to the majority sex group to protect confidentiality

^c^Worst injury method-ICD-based Injury Severity Score less than or equal to 0.941 considered as serious injury

^d^SEIFA used in this study is the Index of Relative Socio-Economic Advantage and Disadvantage (IRSAD), are state deciles based on Statistical Local Areas (SLAs). A low score indicates relatively greater disadvantage. For further information refer http://www.abs.gov.au/ausstats/abs@.nsf/lookup/2033.0.55.001main+features100042011

< 5 Cells with frequency less than 5 or statistics based on less than 5 patients has been suppressed for confidentiality reasons. # Other cells may also be suppressed to protect the confidentiality of < 5 cells

In-hospital death occurred in 0.9% of the patients during the index admission (includes all statistical separations and transfers within the episode). Patients had on average 2.4 complications (CI 2.4–2.5) with a median of 2 (IQR, 1–3); more than one in four (26.8%) needed care post-discharge from hospital. The mean admission cost of patients was AUD 7013 (CI AUD 6929-AUD 7096); cost data were available only for 77.6% of the 152,835 patients. The median LOS was one day (IQR 1–3). ICU stay was recorded in only 3.0% of patients, while 1.5% used the mechanical ventilator and 0.4% required CCU stay. At least one all-cause readmission occurred in 12.3% of patients, non-planned readmission in 7.9% and a potentially avoidable readmission in 3.2% (Table 2).

**Table 2:** Description of injury outcomes by population groups

	Outcomes					
	In-hospital death, n (%)^a^	Average number of CHADx complications^b^, mean (CI)	Care needed post index admission discharge^c^, n (%)	Hospital cost of index admission^d,a^ (A$), mean (CI)		Length of stay of index admission (days), median (IQR)
No of patients selected for analysis	152,795	152,835	148,539	118,570		152,835
No of patients with outcome	1341	20300^e^	39,731	118,570		152,835
Overall indicator	0.9%	2.4 (2.4–2.5)	26.8%	7013 (6929–7096)		1 (1–3)
Age group (years)						
0–14 years	9 (0.0)	1.5 (1.4–1.6)	7451 (35.5)	3221 (3092–3350)		1 (1–1)
15–24 years	13 (0.1)	1.8 (1.7–1.9)	5282 (23.5)	4639 (4476–4802)		1 (1–1)
25–44 years	57 (0.2)	1.9 (1.8–1.9)	7606 (21.9)	5311 (5156–5466)		1 (1–2)
45–64 years	79 (0.3)	2.1 (2.0–2.1)	6318 (21.9)	6864 (6658–7071)		1 (1–3)
65–84 years	459 (1.6)	2.6 (2.6–2.7)	7261 (27.0)	11,484 (11233–11,736)		3 (1–13)
85 and over	724 (4.4)	2.9 (2.9–3.0)	5813 (39.8)	13,380 (13067–13,694)		6 (1–21)
Gender						
Male	671 (0.8)	2.4 (2.3–2.4)	21,698 (26.1)	6336 (6225–6446)		1 (1–2)
Female	670 (1.0)	2.5 (2.4–2.5)	18,033 (27.6)	7889 (7762–8017)		1 (1–5)
Number of comorbidities^f^						
0	430 (0.3)	2.1 (2.1–2.2)	30,797 (25.5)	5373 (5298–5448)		1 (1–2)
1	379 (1.8)	2.7 (2.6–2.7)	5891 (29.6)	10,725 (10447–11,003)		2 (1–10)
2	276 (4.8)	3.0 (2.9–3.1)	1942 (37.6)	17,230 (16497–17,964)		6 (2–20)
3	152 (7.2)	3.2 (3.0–3.3)	745 (39.4)	21,173 (20059–22,288)		10 (4–27)
> 3	104 (11.3)	3.6 (3.4–3.9)	356 (45.6)	29,884 (27596–32,172)		18 (7–36)
Injury severity^g^						
Serious injury (ICISS< 0.941)	945 (5.0)	3.1 (3.0–3.1)	6617 (38.0)	21,019 (20544–21,494)		9 (3–26)
Other injury (ICISS> = 0.941)	396 (0.3)	1.9 (1.9–2.0)	33,114 (25.3)	4988 (4931–5045)		1 (1–2)
Body region						
Head	312 (1.2)	2.5 (2.4–2.6)	5533 (22.1)	4974 (4774–5173)		1 (1–1)
Neck	42 (1.2)	2.6 (2.4–2.9)	630 (19.0)	7879 (6966–8791)		1 (1–3)
Thorax	99 (1.3)	2.5 (2.4–2.6)	1382 (19.4)	8614 (8132–9095)		2 (1–7)
Abdomen, lower back, lumbar spine & pelvis	106 (1.1)	2.4 (2.3–2.5)	2319 (25.6)	8894 (8488–9300)		2 (1–11)
Shoulder & upper arm	74 (0.7)	2.0 (1.9–2.1)	2825 (25.7)	7192 (6938–7445)		1 (1–3)
Elbow & forearm	21 (0.1)	1.7 (1.6–1.7)	4456 (29.2)	5257 (5132–5382)		1 (1–2)
Wrist & hand	7 (0.0)	1.4 (1.3–1.5)	7980 (31.3)	3259 (3206–3312)		1 (1–1)
Hip & thigh	503 (3.9)	3.1 (3.0–3.2)	4623 (39.0)	18,695 (18306–19,084)		8 (2–26)
Knee & lower leg	52 (0.3)	1.9 (1.9–2.0)	4903 (25.1)	9273 (9011–9536)		2 (1–4)
Ankle & foot	7 (0.1)	1.8 (1.6–1.9)	1338 (24.0)	5158 (4881–5436)		1 (1–2)
Foreign body	11 (0.4)	1.8 (1.6–2.1)	617 (24.4)	2879 (2697–3060)		1 (1–2)
Burns	6 (0.3)	2.6 (2.3–2.8)	608 (34.3)	10,265 (8864–11,666)		1 (1–4)
Multiple body regions	0 (0.0)	2.0 (0.9–3.1)	7 (21.2)	3888 (1728–6049)		1 (1–2)
Unspecified body region	2 (0.4)	1.9 (1.4–2.3)	84 (16.2)	4869 (3663–6076)		1 (1–4)
Body region not relevant	99 (0.9)	2.2 (2.1–2.4)	2426 (23.2)	5417 (5148–5687)		1 (1–3)
Grouped injury type (first occurring)						
Superficial injury	32 (0.4)	1.9 (1.8–2.1)	1363 (19.1)	3547 (3371–3723)		1 (1–2)
Open wound	44 (0.2)	1.7 (1.7–1.8)	6065 (29.2)	3352 (3266–3438)		1 (1–4)
Fracture	769 (1.2)	2.6 (2.6–2.6)	19,884 (32.2)	10,096 (9959–10,232)		2 (1–6)
Dislocation, sprain & strain	18 (0.2)	1.6 (1.5–1.8)	1515 (14.5)	4005 (3820–4191)		1 (1–1)
Injury to nerves & spinal cord	11 (0.6)	2.7 (2.3–3.2)	612 (31.2)	11,097 (9125–13,069)		1 (1–2)
Injury to blood vessels	7 (0.5)	1.9 (1.6–2.3)	389 (29.8)	8686 (7363–10,009)		1 (1–2)
Injury to muscle & tendon	5 (0.1)	1.6 (1.5–1.7)	1806 (22.2)	4075 (3923–4227)		1 (1–2)
Crushing injury	0 (0.0)	1.2 (0.8–1.5)	86 (26.5)	2428 (1841–3015)		1 (1–1)
Traumatic amputation	< 5	2.0 (1.7–2.4)	550 (34.8)	5758 (5151–6366)		1 (1–2)
Eye injury- excluding foreign body	< 5	1.9 (1.4–2.3)	227 (48.4)	5862 (4676–7048)		1 (1–3)
Intracranial injury	260 (4.3)	3.0 (2.9–3.2)	1066 (19.0)	9675 (8970–10,380)		1 (1–5)
Injury to internal organs	27 (1.6)	2.7 (2.5–2.9)	431 (26.8)	14,381 (13146–15,616)		4 (2–8)
Foreign body	11 (0.4)	1.8 (1.6–2.1)	617 (24.4)	2879 (2697–3060)		1 (1–1)
Burns	6 (0.3)	2.6 (2.3–2.8)	608 (34.3)	10,265 (8864–11,666)		1 (1–5)
Other and unspecified injury	50 (0.4)	1.8 (1.7–1.9)	2086 (16.4)	3189 (3062–3317)		1 (1–1)
Systemic-poisoning/toxic effects	52 (0.5)	2.1 (2.0–2.3)	2175 (23.2)	4794 (4566–5022)		1 (1–2)
Other effects of external cause/complication	47 (4.0)	2.6 (2.3–3.0)	251 (22.9)	11,259 (9494–13,023)		2 (1–6)
SEIFA						
1	152 (0.9)	2.5 (2.4–2.6)	5142 (32.1)	6990 (6767–7214)		1 (1–3)
2	#	2.4 (2.3–2.6)	#	#		1 (1–3)
3	112 (1.1)	2.5 (2.4–2.6)	2745 (28.4)	7391 (7087–7695)		1 (1–4)
4	154 (0.9)	2.4 (2.3–2.5)	5274 (32.2)	7341 (7081–7602)		1 (1–3)
5	87 (0.9)	2.4 (2.3–2.5)	3057 (30.8)	6412 (6135–6688)		1 (1–2)
6	85 (0.8)	2.3 (2.2–2.4)	3057 (27.9)	6824 (6494–7153)		1 (1–3)
7	75 (0.7)	2.5 (2.4–2.6)	2987 (27.5)	7079 (6780–7377)		1 (1–3)
8	207 (0.8)	2.4 (2.4–2.5)	5156 (21.7)	6736 (6526–6947)		1 (1–3)
9	197 (0.9)	2.4 (2.4–2.5)	5632 (25.6)	7069 (6841–7297)		1 (1–3)
10	195 (0.9)	2.4 (2.4–2.5)	4000 (19.0)	7066 (6816–7315)		1 (1–3)
999	< 5	–	< 5	< 5		< 5
	Outcomes					
	Use of critical care services at index admission n (%)			Readmission^h^, n (%)		
	ICU stay > 0 h	MV use > 0 h	CCU stay > 0 h	30 day-all-cause	30 day -non-planned	30 day-potentially avoidable
No of patients selected for analysis	152,835	152,835	152,835	149,943	149,943	149,943
No of patients with outcome	4519	2234	664	18,514	11,803	4777
Overall indicator	3.0%	1.5%	0.4%	12.3%	7.9%	3.2%
Age group (years)						
0–14 years	158 (0.8)	77 (0.4)	8 (0.0)	1346 (6.4)	856 (4.1)	380 (1.8)
15–24 years	687 (3.0)	418 (1.8)	64 (0.3)	1893 (8.4)	1244 (5.5)	512 (2.3)
25–44 years	1244 (3.5)	780 (2.2)	94 (0.3)	3508 (10.1)	2369 (6.8)	1083 (3.1)
45–64 years	1069 (3.6)	563 (1.9)	143 (0.5)	3627 (12.5)	2182 (7.5)	1000 (3.5)
65–84 years	962 (3.4)	332 (1.2)	222 (0.8)	4992 (18.3)	3032 (11.1)	1123 (4.1)
85 and over	399 (2.4)	64 (0.4)	133 (0.8)	3148 (20.3)	2120 (13.6)	679 (4.4)
Gender						
Male	2645 (3.1)	1429 (1.7)	325 (0.4)	9292 (11.1)	6071 (7.3)	2647 (3.2)
Female	1874 (2.8)	805 (1.2)	339 (0.5)	9222 (13.9)	5732 (8.6)	2130 (3.2)
Number of comorbidities^f^						
0	1936 (1.6)	924 (0.8)	310 (0.3)	13,105 (10.8)	8091 (6.7)	3490 (2.9)
1	1400 (6.7)	746 (3.5)	175 (0.8)	3491 (17.2)	2339 (11.5)	818 (4.0)
2	724 (12.7)	366 (6.4)	106 (1.9)	1172 (22.1)	825 (15.6)	277 (5.2)
3	280 (13.2)	135 (6.4)	42 (2.0)	484 (25.1)	346 (17.9)	124 (6.4)
> 3	179 (19.4)	63 (6.8)	31 (3.4)	262 (32.3)	202 (24.9)	68 (8.4)
Injury severity^g^						
Serious injury (ICISS< 0.941)	2367 (12.4)	1171 (6.1)	192 (1.0)	3364 (18.8)	2366 (13.2)	953 (5.3)
Other injury (ICISS> = 0.941)	2152 (1.6)	1063 (0.8)	472 (0.4)	15,150 (11.5)	9437 (7.1)	3824 (2.9)
Body region						
Head	783 (3.0)	604 (2.3)	87 (0.3)	2654 (10.4)	1894 (7.5)	629 (2.5)
Neck	147 (4.3)	77 (2.2)	13 (0.4)	351 (10.5)	261 (7.8)	93 (2.8)
Thorax	617 (8.4)	224 (3.0)	59 (0.8)	1046 (14.6)	739 (10.3)	227 (3.2)
Abdomen, lower back, lumbar spine & pelvis	346 (3.7)	125 (1.3)	31 (0.3)	1501 (16.3)	1031 (11.2)	354 (3.9)
Shoulder & upper arm	100 (0.9)	27 (0.2)	38 (0.3)	1602 (14.4)	955 (8.6)	432 (3.9)
Elbow & forearm	48 (0.3)	15 (0.1)	47 (0.3)	1925 (12.5)	1069 (7.0)	519 (3.4)
Wrist & hand	27 (0.1)	10 (0.0)	57 (0.2)	2046 (8.0)	1149 (4.5)	572 (2.2)
Hip & thigh	557 (4.3)	81 (0.6)	109 (0.8)	2249 (18.3)	1475 (12.0)	532 (4.3)
Knee & lower leg	147 (0.7)	37 (0.2)	59 (0.3)	2260 (11.5)	1298 (6.6)	703 (3.6)
Ankle & foot	15 (0.3)	6 (0.1)	8 (0.1)	653 (11.7)	441 (7.9)	241 (4.3)
Foreign body	43 (1.7)	21 (0.8)	11 (0.4)	228 (9.0)	143 (5.6)	37 (1.5)
Burns	113 (6.3)	75 (4.2)	7 (0.4)	275 (15.5)	222 (12.5)	138 (7.8)
Multiple body regions	< 5	0 (0.0)	0 (0.0)	7 (21.2)	< 5	0 (0.0)
Unspecified body region	< 5	0 (0.0)	0 (0.0)	81 (15.4)	#	12 (2.3)
Body region not relevant	1573 (14.4)	932 (8.5)	138 (1.3)	1636 (15.6)	1075 (10.2)	288 (2.7)
Grouped injury type (first occurring)						
Superficial injury	56 (0.8)	26 (0.4)	15 9 (0.2)	853 (11.7)	565 (7.8)	157 (2.2)
Open wound	86 (0.4)	43 (0.2)	60 (0.3)	1984 (9.4)	1226 (5.8)	523 (2.5)
Fracture	1431 (2.3)	390 (0.6)	314 (0.5)	8868 (14.2)	5431 (8.7)	2642 (4.2)
Dislocation, sprain & strain	28 (0.3)	< 5	13 (0.1)	902 (8.6)	556 (5.3)	216 (2.1)
Injury to nerves & spinal cord	53 (2.7)	34 (1.7)	5 (0.3)	176 (9.0)	116 (5.9)	47 (2.4)
Injury to blood vessels	49 (3.7)	30 (2.3)	7 (0.5)	107 (8.2)	66 (5.0)	#
Injury to muscle & tendon	#	< 5	13 (0.2)	634 (7.8)	368 (4.5)	162 (2.0)
Crushing injury	0 (0.0)	0 (0.0)	0 (0.0)	27 (8.3)	14 (4.3)	< 5
Traumatic amputation	11 (0.7)	9 (0.6)	< 5	187 (11.8)	103 (6.5)	66 (4.2)
Eye injury- excluding foreign body	< 5	< 5	< 5	92 (19.4)	63 (13.3)	23 (4.8)
Intracranial injury	587 (9.7)	466 (7.7)	38 (0.6)	689 (12.2)	526 (9.3)	181 (3.2)
Injury to internal organs	426 (25.3)	180 (10.7)	18 (1.1)	233 (14.4)	197 (12.2)	72 (4.5)
Foreign body	43 (1.7)	21 (0.8)	11 (0.4)	228 (9.0)	143 (5.6)	37 (1.5)
Burns	113 (6.3)	75 (4.2)	7 (0.4)	275 (15.5)	222 (12.5)	138 (7.8)
Other and unspecified injury	43 (0.3)	20 (0.2)	22 (0.2)	1623 (12.5)	1132 (8.7)	198 (1.5)
Systemic-poisoning/toxic effects	1451 (14.8)	853 (8.7)	126 (1.3)	1483 (15.8)	973 (10.4)	247 (2.6)
Other effects of external cause/complication	122 (10.4)	79 (6.7)	12 (1.0)	153 (13.9)	102 (9.2)	41 (3.7)
SEIFA	614 (3.7)	277 (1.7)	52 (0.3)	1906 (11.8)	1335 (8.3)	554 (3.4)
1	#	#	#	#	#	#
2	395 (4.0)	155 (1.6)	69 (0.7)	1212 (12.4)	816 (8.4)	348 (3.6)
3	539 (3.2)	275 (1.6)	67 (0.4)	2095 (12.6)	1404 (8.5)	538 (3.2)
4	281 (2.8)	150 (1.5)	30 (0.3)	1086 (10.9)	716 (7.2)	302 (3.0)
5	323 (2.9)	184 (1.6)	41 (0.4)	1340 (12.2)	904 (8.2)	394 (3.6)
6	309 (2.8)	152 (1.4)	42 (0.4)	1265 (11.6)	857 (7.8)	350 (3.2)
7	666 (2.7)	359 (1.5)	130 (0.5)	2924 (12.2)	1862 (7.8)	775 (3.2)
8	581 (2.6)	284 (1.3)	115 (0.5)	2849 (12.8)	1711 (7.7)	652 (2.9)
9	526 (2.4)	281 (1.3)	108 (0.5)	2792 (13.1)	1520 (7.1)	598 (2.8)
10	< 5	< 5	< 5	< 5	< 5	< 5
999	614 (3.7)	277 (1.7)	52 (0.3)	1906 (11.8)	1335 (8.3)	554 (3.4)

Note:

^a^Patients whose LOS was > 365 days were excluded

^b^Complications listed in the index admission and related readmissions, includes statistical separations and transfers

^c^Includes separation to private residence/accommodation with support services, inpatient rehabilitation or institutional care, excludes patients who left against medical advice or died in hospital and those who did not originally come from private residence/accommodation

^d^Patients with cost data available in public hospitals only, includes statistical separations and transfers to other hospitals, standardised 2012 prices

^e^Presence of at least one CHADx code

^f^Comorbidities identified using Charlson and Elixhauser algorithms

^g^Worst injury method-ICD-based Injury Severity Score less than or equal to 0.941 considered as serious injury

^h^Eligible index admissions excluded patients who died in hospital or left against medical advice

< 5 Cells with frequency less than 5 or statistics based on less than 5 patients has been suppressed for confidentiality reasons. # Other cells may also be suppressed to protect the confidentiality of < 5 cells

### Factors associated with in-hospital death

In-hospital death increased with age. Compared to patients in the 65–84-year age group, those who were 85 years and over had over two and a half times the odds of death in hospital while death was far less likely among all other age groups. In-hospital death was not notably different for females and males. Likelihood of death increased with the number of comorbidities; patients with more than three comorbidities had thirty-six times the odds of an in-hospital death than those with no comorbidities. Serious injury compared to non-serious injury had a greater impact on in-hospital death (nearly 17 times the odds). Among patients with serious injury, 5.0% died in hospital. Patients with hip & thigh injuries had around 3.4 times the odds of death in hospital than those with head injuries. Apart from hip & thigh, injury to any other body region had a lesser likelihood of death or was not notably different from the likelihood of death from head injuries. Compared to intracranial injury, most other types of injury had more than 30% lower odds of death. There was no notable difference in the odds of in-hospital death among those in more disadvantaged socio-economic areas against more advantaged areas (Tables 2 and 3).

**Table 3:** Associations between injury outcomes and population groups: results of univariate modelling

	Outcomes					
	In-hospital death OR (95% CI)	Number of complications IRR (95% CI)	Care needed post index admission discharge OR (95% CI)	Hospital cost (index admission) Beta coefficient (95% CI)		Length of stay (days) IRR (95% CI)
Age group (years)						
0–14 years	0.03 (0.01–0.05)	0.07 (0.06–0.08)	1.48 (1.43–1.54)	− 0.86 (− 0.89--0.84)		0.13 (0.13–0.13)
15–24 years	0.03 (0.02–0.06)	0.17 (0.16–0.18)	0.83 (0.79–0.86)	− 0.61 (− 0.64--0.59)		0.21 (0.20–0.21)
25–44 years	0.10 (0.07–0.13)	0.21 (0.20–0.22)	0.76 (0.73–0.79)	− 0.53 (− 0.55--0.51)		0.26 (0.26–0.27)
45–64 years	0.16 (0.13–0.20)	0.35 (0.33–0.36)	0.76 (0.73–0.78)	− 0.37 (− 0.39--0.34)		0.40 (0.40–0.41)
65–84 years	Reference	Reference	Reference	Reference		Reference
85 and over	2.78 (2.46–3.12)	1.53 (1.45–1.62)	1.78 (1.71–1.86)	0.17 (0.14–0.19)		1.36 (1.33–1.39)
Gender						
Male	Reference	Reference	Reference	Reference		Reference
Female	1.26 (1.14–1.41)	1.79 (1.73–1.86)	1.08 (1.06–1.11)	0.09 (0.07–0.10)		1.73 (1.71–1.75)
Number of comorbidities						
0	Reference	Reference	Reference	Reference		Reference
1	5.23 (4.55–6.01)	3.26 (3.11–3.41)	1.23 (1.19–1.27)	0.53 (0.51–0.55)		2.59 (2.54–2.64)
2	14.53 (12.46–16.94)	5.80 (5.36–6.27)	1.76 (1.66–1.86)	1.14 (1.10–1.17)		3.99 (3.86–4.12)
3	22.08 (18.25–26.70)	7.76 (6.85–8.79)	1.90 (1.73–2.09)	1.47 (1.41–1.52)		5.00 (4.75–5.27)
> 3	36.16 (28.88–45.28)	10.92 (9.07–13.16)	2.45 (2.13–2.83)	1.94 (1.86–2.02)		6.93 (6.41–7.49)
Injury severity						
Serious injury (ICISS< 0.941)	17.56 (15.60–19.76)	8.83 (8.48–9.19)	1.81 (1.75–1.87)	1.46 (1.44–1.48)		5.08 (4.99–5.17)
Other injury (ICISS> = 0.941)	Reference	Reference	Reference	Reference		Reference
Body region						
Head	Reference	Reference	Reference	Reference		Reference
Neck	1.02 (0.74–1.41)	1.76 (1.58–1.96)	0.82 (0.75–0.90)	0.28 (0.24–0.32)		1.75 (1.67–1.82)
Thorax	1.12 (0.89–1.41)	2.65 (2.45–2.86)	0.85 (0.79–0.91)	0.59 (0.56–0.63)		1.96 (1.90–2.02)
Abdomen, lower back, lumbar spine & pelvis	0.94 (0.75–1.17)	2.71 (2.53–2.91)	1.21 (1.15–1.28)	0.60 (0.57–0.63)		2.52 (2.45–2.59)
Shoulder & upper arm	0.55 (0.42–0.70)	1.38 (1.28–1.48)	1.22 (1.16–1.28)	0.59 (0.56–0.62)		1.42 (1.38–1.46)
Elbow & forearm	0.11 (0.07–0.17)	0.64 (0.59–0.68)	1.45 (1.38–1.52)	0.53 (0.50–0.55)		0.76 (0.74–0.78)
Wrist & hand	0.02 (0.01–0.05)	0.18 (0.16–0.19)	1.60 (1.54–1.67)	0.23 (0.20–0.25)		0.40 (0.39–0.41)
Hip & thigh	3.35 (2.90–3.86)	7.10 (6.68–7.54)	2.25 (2.15–2.36)	1.60 (1.57–1.62)		4.47 (4.36–4.59)
Knee & lower leg	0.22 (0.16–0.29)	1.34 (1.26–1.42)	1.18 (1.13–1.23)	0.92 (0.89–0.94)		1.67 (1.63–1.70)
Ankle & foot	0.10 (0.05–0.22)	0.64 (0.58–0.71)	1.11 (1.04–1.19)	0.34 (0.30–0.37)		0.94 (0.91–0.98)
Foreign body	0.35 (0.19–0.64)	0.45 (0.38–0.53)	1.14 (1.03–1.25)	− 0.04 (− 0.09–0.01)		0.40 (0.38–0.43)
Burns	0.28 (0.12–0.62)	1.94 (1.68–2.23)	1.84 (1.66–2.04)	0.43 (0.38–0.49)		1.71 (1.62–1.81)
Multiple body regions	##	1.11 (0.38–3.23)	0.95 (0.41–2.18)	0.07 (−0.37–0.51)		1.63 (1.10–2.41)
Unspecified body region	0.31 (0.08–1.24)	0.85 (0.63–1.14)	0.68 (0.54–0.86)	0.07 (− 0.05–0.19)		1.26 (1.14–1.40)
Body region not relevant	0.75 (0.60–0.94)	1.18 (1.10–1.27)	1.06 (1.01–1.12)	0.10 (0.07–0.12)		1.01 (0.98–1.03)
Grouped injury type (first occurring)						
Superficial injury	0.10 (0.07–0.14)	0.29 (0.26–0.32)	0.50 (0.47–0.53)	−1.03 (−1.06--1.00)		0.44 (0.43–0.46)
Open wound	0.05 (0.03–0.06)	0.17 (0.16–0.18)	0.87 (0.84–0.90)	− 0.88 (− 0.90--0.86)		0.31 (0.30–0.32)
Fracture	0.27 (0.24–0.31)	Reference	Reference	Reference		Reference
Dislocation, sprain & strain	0.04 (0.02–0.06)	0.20 (0.18–0.22)	0.36 (0.34–0.38)	−0.87 (− 0.90--0.84)		0.27 (0.26–0.28)
Injury to nerves & spinal cord	0.12 (0.07–0.23)	0.42 (0.36–0.49)	0.95 (0.87–1.05)	−0.22 (− 0.27--0.16)		0.72 (0.68–0.76)
Injury to blood vessels	0.12 (0.06–0.25)	0.30 (0.25–0.37)	0.89 (0.79–1.00)	−0.03 (− 0.10–0.04)		0.43 (0.40–0.46)
Injury to muscle & tendon	0.01 (0.01–0.03)	0.16 (0.14–0.17)	0.60 (0.57–0.63)	−0.57 (− 0.61--0.54)		0.26 (0.26–0.27)
Crushing injury	##	0.04 (0.02–0.09)	0.76 (0.59–0.97)	− 1.13 (− 1.28--0.98)		0.18 (0.15–0.21)
Traumatic amputation	0.01 (0.00–0.10)	0.24 (0.20–0.30)	1.12 (1.01–1.25)	− 0.36 (− 0.42--0.29)		0.31 (0.29–0.33)
Eye injury- excluding foreign body	0.05 (0.01–0.33)	0.31 (0.22–0.43)	1.97 (1.64–2.37)	− 0.54 (− 0.64--0.43)		0.39 (0.35–0.44)
Intracranial injury	Reference	1.05 (0.96–1.13)	0.49 (0.46–0.53)	−0.59 (− 0.62--0.56)		1.03 (1.00–1.07)
Injury to internal organs	0.36 (0.24–0.54)	1.64 (1.42–1.89)	0.77 (0.69–0.86)	0.40 (0.34–0.46)		1.04 (0.98–1.10)
Foreign body	0.09 (0.05–0.17)	0.17 (0.14–0.20)	0.68 (0.62–0.75)	− 0.99 (− 1.03--0.94)		0.19 (0.18–0.20)
Burns	0.07 (0.03–0.17)	0.73 (0.63–0.85)	1.10 (0.99–1.21)	− 0.51 (− 0.57--0.46)		0.79 (0.75–0.84)
Other and unspecified injury	0.08 (0.06–0.11)	0.23 (0.22–0.25)	0.41 (0.39–0.43)	− 1.20 (− 1.22--1.18)		0.42 (0.41–0.43)
Systemic-poisoning/toxic effects	0.12 (0.09–0.16)	0.39 (0.36–0.42)	0.64 (0.61–0.67)	−0.91 (− 0.93--0.88)		0.40 (0.39–0.41)
Other effects of external cause/complication	0.92 (0.67–1.27)	0.91 (0.76–1.09)	0.62 (0.54–0.72)	−0.34 (− 0.41--0.27)		1.03 (0.96–1.10)
SEIFA						
1	Reference	Reference	Reference	Reference		Reference
2	1.03 (0.78–1.35)	1.04 (0.95–1.15)	1.08 (1.02–1.14)	0.16 (0.12–0.19)		1.00 (0.96–1.03)
3	1.22 (0.96–1.56)	1.14 (1.04–1.24)	0.84 (0.79–0.89)	0.10 (0.07–0.14)		1.05 (1.02–1.09)
4	0.99 (0.79–1.24)	0.90 (0.83–0.97)	1.00 (0.96–1.05)	−0.04 (−0.07--0.02)		1.04 (1.01–1.07)
5	0.93 (0.72–1.22)	0.83 (0.76–0.91)	0.94 (0.89–0.99)	0.02 (−0.01–0.05)		0.88 (0.85–0.91)
6	0.82 (0.63–1.08)	0.82 (0.75–0.89)	0.82 (0.77–0.86)	−0.03 (− 0.06–0.00)		0.89 (0.86–0.92)
7	0.73 (0.55–0.97)	1.01 (0.92–1.10)	0.80 (0.76–0.84)	− 0.05 (− 0.08--0.02)		1.01 (0.98–1.04)
8	0.92 (0.75–1.14)	0.96 (0.89–1.03)	0.58 (0.56–0.61)	−0.07 (− 0.10--0.04)		0.96 (0.93–0.98)
9	0.95 (0.77–1.17)	1.00 (0.93–1.07)	0.73 (0.70–0.76)	− 0.10 (− 0.13--0.08)		1.04 (1.01–1.07)
10	0.98 (0.79–1.21)	1.02 (0.95–1.10)	0.50 (0.47–0.52)	−0.09 (− 0.12--0.06)		1.04 (1.02–1.07)
999	##	##	##	0.03 (−2.33–2.40)		0.18 (0.02–1.74)
	Outcomes					
	ICU stay OR (95% CI)	MV use OR (95% CI)	CCU stay OR (95% CI)	30 day-all-cause readmission OR (95% CI)	30 day-non-planned readmission OR (95% CI)	30 day-potentially avoidable readmission OR (95% CI)
Age group (years)						
0–14 years	0.21 (0.18–0.25)	0.30 (0.24–0.39)	0.05 (0.02–0.10)	0.31 (0.29–0.32)	0.34 (0.31–0.37)	0.43 (0.38–0.48)
15–24 years	0.87 (0.79–0.96)	1.55 (1.34–1.79)	0.35 (0.26–0.46)	0.41 (0.39–0.43)	0.47 (0.44–0.50)	0.54 (0.49–0.60)
25–44 years	1.02 (0.94–1.11)	1.87 (1.64–2.13)	0.33 (0.26–0.42)	0.50 (0.48–0.52)	0.58 (0.55–0.62)	0.75 (0.69–0.82)
45–64 years	1.06 (0.97–1.16)	1.62 (1.42–1.86)	0.61 (0.49–0.75)	0.64 (0.61–0.67)	0.65 (0.62–0.69)	0.83 (0.76–0.91)
65–84 years	Reference	Reference	Reference	Reference	Reference	Reference
85 and over	0.70 (0.62–0.79)	0.33 (0.25–0.43)	1.02 (0.83–1.27)	1.13 (1.08–1.19)	1.26 (1.19–1.34)	1.06 (0.96–1.17)
Gender						
Male	Reference	Reference	Reference	Reference	Reference	Reference
Female	0.89 (0.84–0.95)	0.71 (0.65–0.77)	1.32 (1.13–1.54)	1.29 (1.25–1.33)	1.21 (1.16–1.25)	1.01 (0.96–1.08)
Number of comorbidities						
0	Reference	Reference	Reference	Reference	Reference	Reference
1	4.46 (4.15–4.78)	4.86 (4.41–5.35)	3.32 (2.76–4.00)	1.72 (1.65–1.79)	1.83 (1.74–1.92)	1.42 (1.31–1.54)
2	9.10 (8.32–9.96)	9.07 (8.01–10.27)	7.50 (6.01–9.37)	2.35 (2.20–2.52)	2.59 (2.39–2.80)	1.87 (1.65–2.12)
3	9.52 (8.33–10.88)	8.99 (7.46–10.83)	8.00 (5.78–11.08)	2.77 (2.49–3.07)	3.06 (2.72–3.45)	2.32 (1.93–2.79)
> 3	15.03 (12.69–17.80)	9.67 (7.43–12.59)	13.74 (9.45–20.00)	3.95 (3.41–4.58)	4.65 (3.96–5.46)	3.10 (2.41–3.98)
Injury severity						
Serious injury (ICISS< 0.941)	8.65 (8.14–9.19)	8.15 (7.49–8.87)	2.87 (2.42–3.39)	1.78 (1.71–1.86)	1.98 (1.89–2.08)	1.88 (1.75–2.03)
Other injury (ICISS> = 0.941)	Reference	Reference	Reference	Reference	Reference	Reference
Body region						
Head	Reference	Reference	Reference	Reference	Reference	Reference
Neck	1.44 (1.20–1.73)	0.97 (0.76–1.23)	1.13 (0.63–2.03)	1.00 (0.89–1.13)	1.05 (0.92–1.20)	1.13 (0.90–1.40)
Thorax	2.95 (2.64–3.28)	1.32 (1.13–1.54)	2.41 (1.73–3.35)	1.47 (1.36–1.58)	1.43 (1.31–1.56)	1.29 (1.10–1.50)
Abdomen, lower back, lumbar spine & pelvis	1.23 (1.08–1.40)	0.57 (0.47–0.69)	0.98 (0.65–1.48)	1.67 (1.56–1.79)	1.57 (1.45–1.70)	1.58 (1.38–1.80)
Shoulder & upper arm	0.29 (0.23–0.36)	0.10 (0.07–0.15)	1.01 (0.69–1.48)	1.45 (1.35–1.55)	1.17 (1.08–1.27)	1.60 (1.41–1.81)
Elbow & forearm	0.10 (0.07–0.13)	0.04 (0.02–0.07)	0.91 (0.64–1.29)	1.23 (1.15–1.31)	0.93 (0.86–1.00)	1.38 (1.22–1.55)
Wrist & hand	0.03 (0.02–0.05)	0.02 (0.01–0.03)	0.66 (0.47–0.92)	0.75 (0.70–0.79)	0.58 (0.54–0.63)	0.90 (0.80–1.01)
Hip & thigh	1.46 (1.30–1.63)	0.27 (0.21–0.34)	2.54 (1.92–3.37)	1.92 (1.81–2.04)	1.70 (1.58–1.82)	1.78 (1.59–2.01)
Knee & lower leg	0.24 (0.20–0.29)	0.08 (0.06–0.11)	0.89 (0.64–1.24)	1.11 (1.05–1.18)	0.88 (0.82–0.95)	1.46 (1.31–1.63)
Ankle & foot	0.09 (0.05–0.14)	0.04 (0.02–0.10)	0.42 (0.20–0.87)	1.13 (1.03–1.24)	1.06 (0.95–1.18)	1.77 (1.52–2.06)
Foreign body	0.54 (0.40–0.74)	0.34 (0.22–0.53)	1.27 (0.68–2.38)	0.85 (0.73–0.97)	0.74 (0.62–0.88)	0.58 (0.42–0.81)
Burns	2.15 (1.76–2.64)	1.82 (1.43–2.33)	1.16 (0.54–2.51)	1.57 (1.38–1.80)	1.78 (1.53–2.06)	3.32 (2.75–4.02)
Multiple body regions	0.92 (0.13–6.72)	##	##	2.31 (1.00–5.32)	1.71 (0.60–4.87)	##
Unspecified body region	0.12 (0.03–0.49)	##	##	1.56 (1.22–1.98)	1.22 (0.90–1.65)	0.92 (0.51–1.64)
Body region not relevant	5.39 (4.94–5.90)	3.91 (3.52–4.34)	3.80 (2.90–4.97)	1.58 (1.48–1.69)	1.42 (1.31–1.53)	1.11 (0.97–1.28)
Grouped injury type (first occurring)						
Superficial injury	0.33 (0.25–0.43)	0.57 (0.39–0.85)	0.41 (0.24–0.69)	0.80 (0.74–0.86)	0.88 (0.81–0.97)	0.50 (0.42–0.59)
Open wound	0.18 (0.14–0.22)	0.33 (0.24–0.45)	0.57 (0.43–0.75)	0.63 (0.60–0.66)	0.65 (0.61–0.69)	0.58 (0.52–0.63)
Fracture	Reference	Reference	Reference	Reference	Reference	Reference
Dislocation, sprain & strain	0.12 (0.08–0.17)	0.05 (0.01–0.14)	0.25 (0.14–0.43)	0.57 (0.53–0.61)	0.59 (0.54–0.64)	0.48 (0.41–0.55)
Injury to nerves & spinal cord	1.19 (0.90–1.57)	2.81 (1.98–4.01)	0.51 (0.21–1.23)	0.59 (0.51–0.69)	0.66 (0.54–0.80)	0.55 (0.41–0.74)
Injury to blood vessels	1.66 (1.24–2.21)	3.73 (2.56–5.43)	1.06 (0.50–2.25)	0.54 (0.44–0.66)	0.56 (0.43–0.71)	0.42 (0.28–0.63)
Injury to muscle & tendon	0.08 (0.05–0.14)	0.06 (0.02–0.18)	0.32 (0.18–0.56)	0.51 (0.47–0.55)	0.49 (0.44–0.55)	0.46 (0.39–0.54)
Crushing injury	##	##	##	0.55 (0.37–0.81)	0.47 (0.28–0.81)	0.21 (0.07–0.66)
Traumatic amputation	0.30 (0.17–0.55)	0.92 (0.48–1.79)	0.25 (0.06–1.02)	0.81 (0.69–0.94)	0.73 (0.60–0.89)	0.98 (0.77–1.26)
Eye injury- excluding foreign body	0.36 (0.14–0.97)	0.68 (0.17–2.72)	0.42 (0.06–2.99)	1.45 (1.15–1.82)	1.60 (1.23–2.09)	1.15 (0.75–1.75)
Intracranial injury	4.67 (4.23–5.16)	13.53 (11.80–15.53)	1.27 (0.91–1.79)	0.83 (0.77–0.91)	1.07 (0.98–1.18)	0.75 (0.64–0.87)
Injury to internal organs	14.69 (13.01–16.59)	19.37 (16.12–23.28)	2.17 (1.35–3.51)	1.02 (0.88–1.17)	1.46 (1.25–1.69)	1.05 (0.83–1.34)
Foreign body	0.73 (0.54–1.00)	1.33 (0.85–2.06)	0.86 (0.47–1.57)	0.59 (0.52–0.68)	0.63 (0.53–0.74)	0.33 (0.24–0.46)
Burns	2.90 (2.38–3.54)	7.03 (5.46–9.04)	0.78 (0.37–1.66)	1.11 (0.97–1.26)	1.50 (1.30–1.73)	1.91 (1.60–2.28)
Other and unspecified injury	0.14 (0.10–0.19)	0.25 (0.16–0.39)	0.34 (0.22–0.52)	0.86 (0.81–0.91)	1.00 (0.94–1.07)	0.35 (0.30–0.40)
Systemic-poisoning/toxic effects	7.56 (7.00–8.16)	15.46 (13.69–17.46)	2.63 (2.13–3.23)	1.13 (1.07–1.20)	1.21 (1.13–1.30)	0.61 (0.53–0.70)
Other effects of external cause/complication	5.02 (4.13–6.10)	11.65 (9.08–14.95)	2.07 (1.16–3.70)	0.97 (0.82–1.15)	1.07 (0.87–1.31)	0.87 (0.64–1.19)
SEIFA						
1	Reference	Reference	Reference	Reference	Reference	Reference
2	0.94 (0.81–1.08)	0.85 (0.69–1.06)	0.39 (0.20–0.76)	1.12 (1.04–1.22)	1.03 (0.93–1.13)	0.97 (0.83–1.12)
3	1.07 (0.94–1.22)	0.93 (0.76–1.13)	2.21 (1.54–3.17)	1.06 (0.98–1.14)	1.01 (0.92–1.11)	1.04 (0.91–1.19)
4	0.85 (0.76–0.96)	0.97 (0.82–1.15)	1.26 (0.88–1.81)	1.08 (1.01–1.15)	1.03 (0.95–1.11)	0.94 (0.84–1.06)
5	0.74 (0.64–0.85)	0.88 (0.72–1.08)	0.94 (0.60–1.48)	0.91 (0.84–0.99)	0.86 (0.78–0.94)	0.88 (0.76–1.01)
6	0.77 (0.67–0.88)	0.98 (0.81–1.18)	1.16 (0.77–1.75)	1.03 (0.96–1.11)	0.99 (0.91–1.08)	1.04 (0.91–1.19)
7	0.74 (0.65–0.85)	0.81 (0.67–0.99)	1.20 (0.80–1.81)	0.97 (0.90–1.05)	0.94 (0.86–1.03)	0.93 (0.81–1.06)
8	0.73 (0.65–0.81)	0.88 (0.75–1.03)	1.70 (1.23–2.34)	1.04 (0.97–1.10)	0.93 (0.87–1.00)	0.94 (0.84–1.05)
9	0.68 (0.61–0.77)	0.75 (0.63–0.88)	1.62 (1.17–2.25)	1.10 (1.03–1.17)	0.92 (0.86–0.99)	0.85 (0.76–0.95)
10	0.65 (0.57–0.73)	0.77 (0.65–0.91)	1.59 (1.14–2.22)	1.13 (1.06–1.20)	0.85 (0.79–0.92)	0.81 (0.72–0.91)
999	##	##	##	##	##	##

Notes

## Omitted from analysis due to zero observations with outcome

### Factors associated with complications

A total of 49,440 complications were identified in this study population. The top four classes of complications were cardiovascular (13.7%), gastrointestinal (12.3%), metabolic disorders (12.1%) and genitourinary (10. 7%) complications. The most common subclasses were electrolyte disorders without dehydration (6.4%), hypotension (5.8%), alterations to mental state (5.4%), constipation (5.1%) and cardiac arrhythmias, conduction disturbances and abnormal heart beat (4.8%) (Table 4).

**Table 4:** Summary of hospital acquired complications among injury patients, Victoria 2012–13 to 2013–14

Number of complications in CHADx classes			CHADx subclasses with the twenty largest number of CHADx codes		
Class	Description	n (%)	Subclass	Description	n (%)
5	Cardiovascular complications	6755 (13.7)	15.2	Electrolyte disorders w/o dehydration	3158 (6.4)
7	Gastrointestinal complications	6058 (12.3)	5.6	Hypotension	2859 (5.8)
15	Metabolic disorders	6005 (12.1)	10.4	Alterations to mental state	2688 (5.4)
9	Genitourinary complications	5279 (10.7)	7.4	Constipation	2512 (5.1)
17	Other complications	5046 (10.2)	5.3	Cardiac arrythmias, conduction disturbances & abnormal heart beat	2350 (4.8)
6	Respiratory complications	3970 (8.0)	7.5	Nausea and Vomiting	2036 (4.1)
10	Hospital-acquired psychiatric states	3686 (7.5)	14.2	Other hospital-acquired anaemia	1687 (3.4)
1	Intra and post procedural complications	2696 (5.5)	9.2	Urinary tract infection	1660 (3.4)
14	Haematological disorders	2605 (5.3)	9.3	Urinary retention	1574 (3.2)
2	Adverse drug events	2584 (5.2)	6.3	Acute lower respiratory infections (including influenza & pneumonia)	1458 (2.9)
8	Skin conditions	2541 (5.1)	8.3	Dermatitis, rash and other skin effects	1398 (2.8)
3	Accidental injuries	955 (1.9)	15.1	Dehydration/volume depletion	1148 (2.3)
4	Specific infections	692 (1.4)	1.7	Other complications of surgical and Medical care not elsewhere classified (including shock)	1063 (2.2)
16	Nervous system complications	551 (1.1)	9.1	Acute & unspecified kidney failure	1028 (2.1)
11	Early pregnancy complications	0 (0.0)	6.1	Adult respiratory distress syndrome (ARDS), respiratory failure and pulmonary collapse (including atelectasis)	981 (2.0)
12	Labour, delivery & postpartum complications	4 (0.0)	8.1	Pressure injury	957 (1.9)
13	Perinatal complications	13 (0.0)	9.4	Other complications and symptoms of the urinary system	940 (1.9)
			2.16	Adverse effects due to other drugs	935 (1.9)
			15.5	Disorders of mineral metabolism	786 (1.6)
			17.6	Fever (not classified to condition)	769 (1.6)
				All other CHADx subclasses	17,453 (35.3)

Note: Total number of CHADx conditions =49,440

The average number, and odds of occurrence of complications increased with increasing age and with increasing number of comorbidities. The odds of complications were higher for patients with serious injury. The likelihood of complications varied by body region, with injuries to neck, thorax, shoulder and upper arm and some lower extremities incurring higher likelihood of complications than head injuries. Complications were more likely to occur in those with injury to internal organs (64% higher odds) compared to those with fractures (Tables 2 and 3). There was no notable difference in likelihood of complications between socioeconomic areas.

### Factors associated with discharge destination

More than one-fifth of patients required further support services after discharge from hospital. This was observed in all age groups, with higher odds in children and those 85 years and over compared to the reference group (65–84 years). The impact of gender did not stand out (females 8% higher odds than males) while more severe injuries and increasing number of comorbidities increased the odds of needing support services. Patients with upper limb and hip and thigh injuries were more likely to require further care compared to head injuries. Patients with eye injuries were more likely to require further care compared to fractures. The likelihood of being discharged to care with support services decreased with increase in socioeconomic advantage (Tables 2 and 3).

### Factors associated with cost

The cost of treating patients increased with increasing patient age, number of comorbidities, injury severity and female gender. Severe injuries and increasing number of comorbidities increased the likelihood of higher costs. The highest likelihood of increased costs were for knee and lower leg and hip and thigh injuries compared to head injuries, while injuries to most other body parts were also likely to increase costs compared to head injuries. Compared to fractures, injuries to internal organs were more likely to increase costs while all other injury types were likely to result in lower costs or did not produce a notable difference. There was no notable difference in likelihood of incurring higher costs between socioeconomic areas (Tables 2 and 3).

### Factors associated with LOS

The median LOS (in days) was greater for those aged 65–84 (3, IQR 1–13) and 85 years and over (6, IQR 1–21) as opposed to the younger age groups (1, IQR 1–3). Relative to the 65–84 years age group, those 85 years and above were 36% more likely to have a longer LOS and those in the younger age groups were around 60–87% less likely. The mean LOS was 5.4 days (not shown in tables). Although the gender difference in median LOS was not notable, the mean was 4.1 days for males and 7.1 days for females (not shown in tables). Severe injuries and more comorbidities were associated with greater LOS. Injuries to the neck, trunk, shoulder and upper arm and lower extremities compared to the head had higher incident rates. Fractures had higher incident rates compared to most other types of injuries. There was no notable difference in incident rates between socioeconomic areas (Tables 2 and 3).

### Factors associated with CCS use

ICU stay and MV use proportions and the likelihood of using these services was highest among those aged 25–44 years and 45–64-year age group, while CCU use was greater among the 65–84-year and 86 years and over age groups. Females were more likely to stay in the CCU but gender was not statistically significantly with ICU and MV use. Severe injuries and comorbidities were associated with increased likelihood of CCS use. The body region injured and the type of injury affected the likely use of CCSs. The likelihood of ICU-stay and MV use were less as the socioeconomic advantage increased while it was the opposite for CCU stay (Tables 2 and 3).

### Factors associated with readmission

The proportions of cases with 30-day readmissions increased with increasing age, injury severity and number of comorbidities. Potentially-avoidable readmissions within 30 days were equally common among males and females than the other two types of readmissions, which were more common among females. The likelihood of readmission was notably lower in the 0–14 and 15–24-year-old age groups compared to the 65–84-year (reference) age group for all three types of readmissions, while the 25–44- and 45–64-year age groups also had a notably lower likelihood of all-cause and non-planned readmissions compared to the 65–84-year (reference) age group. The effect of gender was not notable among all three types of readmissions while severe injuries were more than 70% more likely to result in any type of readmission. Increasing number of comorbidities increased the likelihood of all three types of readmissions. Injuries to the trunk and hip and thigh and burns to some body regions were more likely to result in any type of readmissions compared to the head, while shoulder and upper arm injuries were more likely to result in all-cause and potentially avoidable readmissions. Knee and lower leg and ankle and foot injuries were also more likely to result in potentially avoidable readmissions. Compared to fractures, eye injuries were more likely to result in all-cause readmissions and non-planned readmissions, while injury to internal organs were more likely to result in non-planned readmissions. Burns were more likely to result in both non-planned and potentially avoidable readmissions compared to fractures. There was no notable difference in likelihood of 30-day readmissions between socioeconomic areas (Tables 2 and 3).

### Associations between outcomes

Many of the outcomes were associated with each other. The results and a discussion of this analysis is provided as an appendix with supporting Additional file 1: Tables S1 and S2, as the main focus of this study was to establish the associations between base factors (mostly those present at point of hospital admission) and outcomes.

## Discussion

This study described injury outcomes in terms of mortality, complications and burden of hospital-admitted injury patients in Victoria. These outcomes are shown to differ by age, gender, comorbidities, body region injured, injury type and injury severity.

The main finding is that outcomes and risk factors differ depending on the outcome measured, and the method used to measure the outcome. Increasing age was associated with all outcomes. The need for post hospital-discharge care on the other hand was highest among children followed by the 85 and over age group. MV use was more likely to be by those in the 15–64 age group than the 65 and above age group. Gender was only likely to effect complications, LOS and CCU stay (females more likely than males). Severe injuries increased the likelihood of all adverse outcomes. Increasing number of comorbidities increased the likelihood of all adverse outcomes. Injuries to certain body regions had an increased likelihood of negative outcomes; body regions with notably increased likelihoods of adverse effects varied by outcome. Injury to internal organs, intracranial injury, burns, systematic poisonings, eye injuries and injury to blood vessels were likely to increase adverse outcomes. Comparing outcomes, determining demographic and injury-related factors associated with adverse outcomes and thereby identifying groups at risk can help target secondary and tertiary injury prevention measures.

Numerous other studies [7, 10, 14] have treated age, gender and injury severity as confounders and the findings from our analysis confirm that age and injury severity are risk factors for most of the outcomes measured in this population. Overall, results on age, gender, body region and injury type proportions, LOS and injury severity aligned with previous Australian national statistics [1, 2]. The proportion of injury patients with comorbidities in this study was 19.5% as opposed to a previous research for New South Wales (NSW), Queensland and South Australia [58] which was 15.6%; the difference is likely to be attributable to the fact that this study included 31 conditions as opposed to the 17 CCI conditions used in the other study.

### In-hospital death

Our finding that increasing age, injury severity and comorbidities are associated with mortality after injury has been previously documented [5]. The proportion of patients who died in hospital (0.9% of index admissions) in this study is not dissimilar to a reported national proportion of 0.97% for 2005–06, though the latter was not limited to injury and contained more exclusions than ours (excluded palliative care and neonates) [59]. The proportion of 5.0% in-hospital deaths among serious injury patients in this study is close to the 4.4% reported for a severe trauma population for Victoria using data from 1996 to 2001 [21]. These two findings imply that injury patients have a higher rate of in-hospital death over general hospital admitted patients (includes injury and non-injury) and greater injury severity increases the risk. The proportion of deaths-in-hospital for the 85 years and over group (4.4%) is lower than previously published research of 9.5% for a cohort of patients admitted to an academic trauma centre in USA between 2006 and 2010 [17]. The latter cohort consisted of patients 90 years and over, the difference in the age cut-off most likely to contribute to the difference.

Intracranial injury was more likely to result in in-hospital death compared to other injury types, which echoes findings from previous work [17]. This study found that comorbidities had a notable effect on the outcome. There is varied findings on the effect of comorbidity on mortality among injury patients. Some studies also argued that the effect of increasing age and serious injury dominate the outcome over the presence of comorbidities [5, 11, 14, 20, 21, 23, 35, 60]; in other words, these studies [11, 14, 21, 35, 60] concluded that once age and injury severity were adjusted for, the effect of comorbidity was not significant, while others [5, 20, 23] concluded that the effect of comorbidity was significant among patients with minor or moderately severe injuries. The majority of injuries, however, are not severe, and therefore assessing the effects of comorbidity among mild to moderately injured patients is still valuable.

### Complications

The findings from this study that age, comorbidities and injury severity increase the likelihood of complications confirms findings of previous research [10]. This study also found associations between body region and injury type with complications. The finding here is novel in revealing information on the effect of patient characteristics on hospital acquired complications for injury patients: increasing age, injury type and severity, and comorbidities all increased the likelihood of hospital-acquired complications.

### Discharge destination

This study confirms the finding that injury severity and comorbidities are likely to influence discharge destination [9, 10, 12, 15], but found that gender had no effect; contradicting a finding from a previous study [36]. The difference can be attributed to the fact that the study by Brown et al. (2012) [36] was based on a group of traumatic brain injury patients aged 65 years and over and they categorised patients discharged home with support services as not needing long term care, contrary to our categorisation. They also adjusted for marital status and ethnicity. We found older females to have a higher proportion of being discharged with support services over their male counterparts which could partly explain the findings in the former study. The exclusion of patients who originally came from long-term care institutions has reduced the number of patients needing further care. However, the proportion of such patients was less than 2%; therefore, the impact on the results would have been relatively minor.

### Cost

The finding that hospital admission costs for injury patients are associated with injury severity has been reported in previous studies [14, 18]. These studies also found age to be associated with increased costs. The association of costs with comorbidity has also been established previously [14]. Findings from this study that costs can be higher for patients with lower extremity injuries and injury to blood vessels and internal organs have not been documented in past research. Injuries to lower extremities limit mobility and therefore delay discharge from hospital. This can be observed in the extended LOS for patients with hip and thigh injuries. The extended LOS and possibly major surgeries could be attributable to higher costs for this group of patients. The higher costs associated with injury to blood vessels and internal organs could be attributable to the fact that some of the patients with such injuries were the most likely to have an ICU stay and use the mechanical ventilator.

### LOS

Age, injury severity and number of comorbidities were found to be associated with LOS in this study and previous research [14, 35]. Injuries to the lower extremities and trunk were likely to increase LOS, which was confirmed in a former study [14]. The mean hospital LOS of 5.4 days in this Victorian population was higher than the national mean of 3.6 days as reported by Pointer (2018) [2] for injury patients in 2014–15, and the current study does corroborate the finding from Pointer (2018) that females are likely to stay longer in hospital. The longer length of stay in the current study was due to the capture of days in hospital for all subsequent statistical separations and inward transfers due to complications within the index admission. The national report on the other hand picks up all records with an injury in the principal diagnosis but does not identify subsequent statistical separations and transfers as part of one episode. These extra records were captured by the use of data linkage in this study. This reveals that data linkage can provide more accurate estimates of burden. The median LOS of 3–6 days among those aged 65 year and over in the present study aligns well with another study in NSW [18] which reported 5–7 days, the higher LOS in the latter could be explained by the fact that it was solely based on level I trauma centre data.

### CCS

Our findings that age, injury severity, comorbidities and body region are associated with ICU and ventilator use was seen in previous work [10, 11, 61]. We did not find any resources with details on CCU use of injury patients to compare, and the low proportion (0.4%) could be the reason that most studies did not focus on this outcome. Factors associated with CCS use are quite different to other outcome measures such as death, cost, LOS and 30-day readmissions. For example, ICU stay or the use of the MV is relatively more common in the 15–64-year age groups whereas the other outcomes are high among the 65 and over age groups. The effect of certain injury types (i.e., intracranial injuries and injuries to internal organs, blood vessels and nerves and spinal cord) is greater on ICU stay and MV use than on other outcomes.

### Readmission

This study’s finding that age, injury severity and comorbidity are associated with 30-day readmissions is aligned with previous research. The non-planned 30-day readmission proportion of 7.9% in this population is a little higher compared to previous findings of 5.9% for trauma patients aged 16 years and above from a Canadian Provincial Trauma system between 1998 and 2009 [19]. For potentially-avoidable 30-day readmissions, the SQLape methodology was utilised in this study. Potentially-avoidable 30-day readmissions are defined as unforeseen readmissions due to a previously known affection under this methodology. These generally consists of (i) iatrogenic complications, (ii) conditions generally resulting from pre-existing conditions and (iii) preventable diseases like deep vein thrombosis, pulmonary embolism and decubitus ulcer [43]. Since pre-existing conditions (comorbidities) were a factor for consideration in this study, patients with a 30-day readmission outcome with a primary diagnosis indicating it was a complication due to a pre-existing condition were not included. This could have contributed to the drop in this outcome (it has been shown that this quotient of patients account for nearly 80% of potentially-avoidable readmissions [43]). Therefore, this study’s use of a limited component of SQLape methodology may not be sufficient to capture the outcome. Finally, even if this component was included, the results may not have been accurate, as per the discussion by Walraven et al., (2011) [62]. They undertook a systematic review of literature on potentially-avoidable readmissions which included the SQLape methodology and stated that though many methods exist for identifying these readmissions, the methods had deficits and the proportions computed using the various methods varied significantly. They concluded that the true proportions of these potentially avoidable readmissions are still unclear.

There is evidence to suggest inter-relationships between outcomes exist; especially the effect of complications on readmissions [19], complications on LOS [33], ventilator use on mortality and cost and LOS [14] and ICU use on readmissions [63]. This study established all the associations mentioned except the association between complications and readmissions which were not significant in this instance. Apart from those noted here, many other associations were also established. Establishing inter-relationships between eleven outcomes is a study strength and this information will be useful in tertiary prevention. For e.g., complications are related to MV use, therefore precautions can be taken to mitigate complications for patients on the MV. They are also useful in research: one outcome may be used as proxy for another. e.g. LOS as proxy for cost, when cost cannot be measured.

### Limitations

Some limitations are noted in this study. We were unable to censor out-of-hospital deaths when selecting 30-day readmission cases, which may have resulted in an under-estimate in 30-day readmissions. We were also unable to estimate the hospital treatment costs for all the patients in the cohort; data on treatment costs were unavailable for private hospitals patients and missing for some of the public hospital patients (around one-fifth in total). Since the discussion of costs in this study is around averages as opposed to totals, we expect that the missing public hospital data would only have a minor impact on the overall conclusions.

Analysis of hospital admissions data does not provide information on all prevalent comorbidities. Coding has relevance for hospital reimbursement, and mostly, only conditions that had an impact on the patient management of the present episode are coded [39]. Validation studies have found that more serious and life-threatening conditions (e.g., cancer) are more likely to be captured in the codes than clinically non-specific and symptomatic conditions (e.g., rheumatologic) [22]; furthermore, coding can be insensitive towards certain conditions such as dementia [64]. Conversely, minor conditions are more likely to get coded among healthier patients as there are no serious ailments to record. Apart from that, in the case of fatalities or long hospital stay, more comorbid conditions are recorded, resulting in higher ORs. Prevalence of comorbidities have been found to be lower in administrative data than in medical records [65] and their clinical severity not recorded [22]. We acknowledge that the use of the U78-U88 codes (supplementary codes for chronic disease identification) incorporated into the VAED since 2015/16 will add value to this work and we propose using these in further research.

The number of complications identified in this study is not a perfect estimate of the total due to: (1) limitations in using hospital administrative databases such as the VAED and (2) limitations of CHADx. Regarding (1): a previous study on the VAED [66] revealed that only 76.2% of admissions were correctly allocated a complication in the ‘condition onset’ flag, which means that this study could be failing to capture about a quarter of the complications. Regarding (2), the drawback of the CHADx: although it aims to minimise double counting of complications, it has been shown to be less than perfect [67], due to the linear representation of conditions in the diagnosis codes, leaving the possibility for some overestimation of CHADx conditions [67]. For the purpose of providing an overview of injury outcomes in terms of complications, however, we considered the CHADx to be adequate.

Further information on the cause of readmission in the VAED would enable better classification of 30-day readmissions. A former study found that most of the readmissions were due to comorbidity than the index primary diagnosis [26], which states that paying attention to the acute condition of the index disease during the post-discharge period can mar the observation /treatment of comorbidities, resulting in readmissions.

Injury outcomes studies can benefit from including other factors such as marital status [68], ethnicity [16, 69] and insurance status [17]. However, we considered these variables to be non-essential for this descriptive study and therefore they were excluded due to space limitations. Further information on the available facilities and capacities, and the location of the individual hospitals (travel time to get access) can also add value to the prediction of outcomes as these features are likely to contribute. Apart from these, a major limitation of predefined databases is that not all the variables required for a study may be readily available.

## Conclusions

This study provides a comprehensive overview of outcomes for hospital-admitted injury patients along with their demographic and injury characteristics; the use of linked data has the advantage of better and longer patient follow-up in administrative data sets.

Similar outcomes when measured in different ways lead to very different results. Most notably, 30-day readmission proportions are higher when considering all-cause 30-day readmission than when considering the non-planned. Proportion of patients requiring mechanical ventilation is half the proportion admitted to the ICU. The magnitude of the effect of certain factors vary between similar outcomes based on how the outcome was measured: the matrix of outcomes vs factors provides meaningful insight into the specific effects of certain patient or injury characteristics on outcomes.

## Additional file

Additional file 1: Associations between outcomes. **Table S1.** Associations between outcomes: results of univariate modelling. **Table S2.** Univariate model types used to output Table S1. (DOCX 16 kb)

## Data Availability

The data that support the findings of this study are available from CVDL but restrictions apply to the availability of this data, which were used under license for the current study, and so are not publicly available. The authors are not in a position to release the data (in accordance with the agreement with CVDL).

## Abbreviations

AUD: Australian dollars
CCI: Charlson Comorbidity Index
CCS: Critical care services
CCU: Cardiac care unit
CHADx: Classification of Hospital Acquired Diagnoses
CI: Confidence interval
CVDL: Centre for Victorian Data Linkage
ECM: Elixhauser Comorbidity Measure
ICD-10-AM: International Statistical Classification of Diseases and Related Health Problems, Tenth Revision, Australian Modifications
ICISS: International Statistical Classification of Diseases based Injury Severity Score
ICU: Intensive care unit
IQR: Inter-quartile range
IRR: Incident rate ratio
IRSAD: Index of Relative Socio-Economic Advantage and Disadvantage
LOS: Length of hospital stay
MV: Mechanical ventilator
NSW: New South Wales
OR: Odds ratio
SEIFA: Socio-Economic Indexes for Areas
SLA: Statistical Local Areas
SQLape: Striving for Quality Level and Analyzing of Patient Expenses
SRR: Survival risk ratio
VAED: Victorian Admitted Episodes Dataset
VCDC: Victorian Cost Data Collection
