# Supplementary material for: Complications, burden and in-hospital death among hospital treated injury patients in Victoria, Australia: a data linkage study
Source: BMC Public Health. 2019 Jun 21;19:798. doi: 10.1186/s12889-019-7080-y (PMC6588941; doi:10.1186/s12889-019-7080-y)
Supplement: Supplementary file 1 — Associations between outcomes. Table S1. Associations between outcomes: results of univariate modelling. Table S2. Univariate model types used to output Table S1. (DOCX 16 kb) [file 12889_2019_7080_MOESM1_ESM.docx]

# Additional file 1

# Associations between outcomes

In-hospital death was likely associated with complications and the use of CCS. Complications were associated with LOS and MV use, which proves what is already known in clinical care. Patients requiring ICU stay or MV use were likely to be discharged with support services. Costs were higher for patients with complications, used CCS or had an in-hospital death. Complications, ICU stay and MV use were likely to increase LOS. Complications and CCU stay were likely to result in an ICU stay or MV use. Complications were also associated with CCU stay. Patients discharged with support services were more likely to have a 30-day readmission against those who were discharged without support services (Table S1). Table S2 presents the model types employed to test significance of associations.

Table S1: Associations between outcomes: results of univariate modelling

| Dependent  Independent | In-hospital death | CHADx complications | Care needed post index admission discharge | Hospital cost of index admission | Length of stay of index admission | ICU stay | MV use | CCU stay | 30 day-all-cause readmission | 30 day -non-planned readmission | 30 day-potentially avoidable readmission |
| --- | --- | --- | --- | --- | --- | --- | --- | --- | --- | --- | --- |
| In-hospital death | - | - | - | 1.51 (1.44-1.58)* | 1.10 (1.03-1.17) | - | - | - | - | - | - |
| CHADx complications | 1.50 (1.48-1.53)* | - | 1.23 (1.22-1.24) | 0.46 (0.45-0.46)* | 0.58 (0.57-0.58)* | 1.60 (1.58-1.62)* | 1.52 (1.50-1.55)* | 1.31 (1.27-1.34)* | 1.20 (1.18-1.21) | 1.21 (1.19-1.22) | 1.23 (1.21-1.24) |
| Care needed post index admission discharge | - | - | - | - | - | - | - | - | 1.76 (1.71-1.82)* | 1.60 (1.54-1.67)* | 1.73 (1.63-1.84)* |
| Hospital cost of index admission | - | - |  | - | - | - | - | - | 1.04 (1.03-1.06) | 1.10 (1.08-1.12) | 1.06 (1.03-1.08) |
| Length of stay of index admission | - | 0.04 (0.04-0.04)* | 1.02 (1.02-1.02) | 0.06 (0.06-0.06) | - | - | - | - | 1.01 (1.01-1.01) | 1.01 (1.01-1.01) | 1.01 (1.01-1.01) |
| ICU stay> 0 hours | 11.16 (9.81-12.69)* | - | 1.71 (1.60-1.83)* | 1.81 (1.77-1.85)* | 1.50 (1.46-1.53)* | - | - | - | 1.83 (1.69-1.98)* | 2.07 (1.89-2.27)* | 1.88 (1.64-2.15)* |
| MV use > 0 hours | 14.88 (12.80-17.29)* | 2.16 (2.12-2.21)* | 1.86 (1.69-2.04)* | 2.04 (1.99-2.09)* | 1.65 (1.60-1.71)* | - | - | 3.56 (2.50-5.07)* | 1.83 (1.63-2.05)* | 2.04 (1.79-2.32)* | 1.97 (1.62-2.38)* |
| CCU stay> 0 hours | 2.81 (1.71-4.64)* | - | 0.90 (0.75-1.08) | 0.97 (0.85-1.09)* | 1.00 (0.90-1.10) | 7.80 (6.41-9.50)* | 3.56 (2.50-5.07)* | - | 1.78 (1.47-2.17)* | 1.95 (1.56-2.44)* | 1.50 (1.04-2.17)* |
| 30 day-all-cause readmission | - | - | - | - | - | - | - | - | - | - | - |
| 30 day -non-planned readmission | - | - | - | - | - | - | - | - | - | - | - |
| 30 day-potentially avoidable readmission | - | - | - | - | - | - | - | - | - | - | - |

Notes: * Significant associations between two outcomes (effect size of 30% or more)

Table S2: Univariate model types used to output Table A1

| **Outcome vs outcome** | **Outcome type - regression model (test statistic)** |
| --- | --- |
| In-hospital death, CCS use, readmissions and discharge destination modelled with each other | Binary vs binary - logistic (OR) |
| In-hospital death /discharge destination vs complications | Binary vs continuous - logistic (OR) |
| CCS use vs complications | Binary vs continuous - logistic (OR) |
| Readmissions vs complications | Binary vs continuous - logistic (OR) |
| Discharge destination vs LOS | Binary vs continuous - logistic (OR) |
| Readmissions vs LOS | Binary vs continuous - logistic (OR) |
| Cost vs in-hospital death/CCS use | Continuous vs binary - log-linear (Beta coefficient) |
| Costs vs complications/LOS | Continuous vs continuous - log-linear (Beta coefficient) |
| LOS vs in-hospital death/CCS use | Continuous vs binary - negative binomial (IRR) |
| LOS vs complications | Continuous vs continuous - negative binomial (IRR) |
| Readmissions vs ln(cost) | Binary vs continuous - logistic (OR) |
| Complications vs LOS | Continuous vs continuous - linear (Beta coefficient) |
| Complications vs MV use | Continuous vs binary - linear (Beta coefficient) |
